# Spatial patterns of bacterial and archaeal communities along the Romanche Fracture Zone (tropical Atlantic)

**DOI:** 10.1111/1574-6941.12142

**Published:** 2013-05-16

**Authors:** Itziar Lekunberri, Eva Sintes, Daniele Corte, Taichi Yokokawa, Gerhard J Herndl

**Affiliations:** 1 Department of Marine Biology, Faculty Center of Ecology, University of ViennaVienna, Austria; 2 Center for Marine Environmental Studies (CMES), Ehime UniversityMatsuyama, Japan; 3 Department of Biological Oceanography, Royal Netherlands Institute for Sea Research (NIOZ)Texel, The Netherlands; 4 Centre for Ecological & Evolutionary Studies (CEES)Groningen, The Netherlands

**Keywords:** Bacteria, catalyzed reporter deposition-fluorescence *in situ* hybridization, deep sea, prokaryotic communities, Romanche Fracture Zone, *Thaumarchaeota*

## Abstract

The composition of prokaryotic communities was determined in the meso- and bathypelagic waters funneled through the Romanche Fracture Zone (RFZ, 2°7′S, 31°79′W to 0°6′N, 14°33′W) in the tropical Atlantic. Distinct water masses were identified based on their physical and chemical characteristics. The bacterial and archaeal communities were depth-stratified with a total of 116 and 25 operational taxonomic units (OTUs), respectively, distributed among the distinct water masses as revealed by terminal restriction fragment length polymorphism, and cloning and sequencing. The relative abundance of *Thaumarchaeota*, determined by catalyzed reporter deposition-fluorescence *in situ* hybridization, was significantly higher in deeper layers (Antarctic Bottom Water, AABW, > 4000 m depth), contributing up to 31% to the total prokaryotic community, than in the mesopelagic and lower euphotic layer. Although the contribution of SAR11 to bacterial abundance did not increase with depth, SAR202, SAR324, SAR406 and *Alteromonas* did increase with depth. Terminal restriction fragment length polymorphism analysis revealed successional changes in the bacterial and archaeal community composition of the North Atlantic Deep Water (NADW) with a passage time through the RFZ of *c*. 4 months but not in the under- and overlying water masses. Our results indicate that specific water masses harbor distinct bacterial and archaeal communities and that the prokaryotic community of the NADW undergoes successional changes in this conduit between the western and eastern Atlantic basin. Apparently, in the absence of major input of organic matter to specific deep-water masses, the indigenous prokaryotic community adapts to subtle physical and biogeochemical changes in the water mass within a time frame of weeks, similar to the reported seasonal changes in surface water prokaryotic communities.

## Introduction

Prokaryotes represent an important component of the marine plankton, constituting up to 70% and 75% of the total biomass in surface and deep waters, respectively (Fuhrman *et al*., 19). Generally, prokaryotic abundance and activity decrease by one to two orders of magnitude from the euphotic to the bathypelagic realm of the ocean (reviewed in Arístegui *et al*., 5). Also, the distribution pattern of the main prokaryotic groups varies considerably with depth. Although *Bacteria* are dominant in the euphotic layer, the contribution of *Thaumarchaeota* to total prokaryotic abundance generally increases with depth in the global ocean (Karner *et al*., 27; Church *et al*., 8; Teira *et al*., 49). Several physical and chemical parameters co-vary with depth, contributing most likely to the depth-stratification of prokaryotic communities (DeLong *et al*., 15; Galand *et al*., 20). Using 454 pyrosequencing, Agogué *et al*. (1) showed that a considerable fraction of the bacterial community is present throughout the water column, whereas some operational taxonomic units (OTUs) are specific for distinct water masses, leading overall to a water mass-specific clustering of bacterial communities in the dark realm of the boreal to tropical Atlantic.

Fracture zones in the Mid-Atlantic Ridge are generally sites of intense mixing of individual water masses that play a major role in the exchange of deep water between the western and eastern basin of the Atlantic (Ferron *et al*., 17). One of the major conduits of Antarctic Bottom Water (AABW) entering the eastern basin of the Atlantic is the Romanche Fracture Zone (RFZ) in the equatorial Atlantic (Mercier & Speer, 33). In terms of water mass transport, the RFZ is the largest and deepest fracture zone in the Mid-Atlantic Ridge, exceeding 7700 m depth in its central part (Mercier & Speer, 33). Through the RFZ, 0.14 Sv (1 Sv = 10^6^ m^3^ s^−1^) of the western North Atlantic Deep Water (NADW) and 1.4 Sv of AABW flow from the western into the eastern Atlantic Basin, while part of the Antarctic Intermediate Water (AAIW) leaves the eastern basin of the Atlantic (Mercier & Speer, 33). Originally, the NADW is a mixture of Iceland-Scotland Overflow Water (ISOW) and Labrador Sea Water (80%) flowing southwards in a western and an eastern branch (van Aken, 2). Part of the western branch of the NADW enters the eastern Atlantic Basin through the RFZ (van Aken, 2). AABW is formed in the Weddell Sea during winter, flows northwards in the western basin of the South Atlantic and eventually enters the eastern basin of the Atlantic, mainly through the RFZ (van Aken, 3).

The aim of our study was to characterize the composition of the bacterial and archaeal communities in the different water masses of the RFZ down to 7700 m depth. We hypothesized that successional changes occur in the prokaryotic community composition of the individual deep-water masses during their passage through the RFZ. The bidirectional flow of water masses through the narrow RFZ enables determination of the transit time of the deep-water masses in the RFZ. For the NADW and the AABW, it takes about 4 and 7 months, respectively, to pass through the RFZ (Mercier & Speer, 33). During the passage of the deep-water masses through the RFZ, changes in the biogeochemical composition of the deep-water masses due to biotic activity might alter the conditions for individual members of the prokaryotic community within specific water masses. We hypothesized that these successional changes in the individual water masses of the RFZ are most pronounced in the NADW, whereas the water mass above the NADW, i.e. the AAIW, is influenced by sedimenting organic matter from the overlying waters, preventing the detection of successional changes in prokaryotic community composition there. In contrast, the AABW underneath the NADW is characterized by very low biogeochemical transformation rates and microbial activity, also preventing the detection of successional changes in the prokaryotic community. To address these questions, we used terminal restriction fragment length polymorphism (T-RFLP), clone libraries and sequencing, fluorescence *in situ* hybridization (FISH) and catalyzed reporter deposition-fluorescence *in situ* hybridization (CARD-FISH) to obtain a detailed view of the prokaryotic community structure and its changes in the individual water masses of the RFZ.

## Materials and methods

### Sampling site

**Figure 1:**
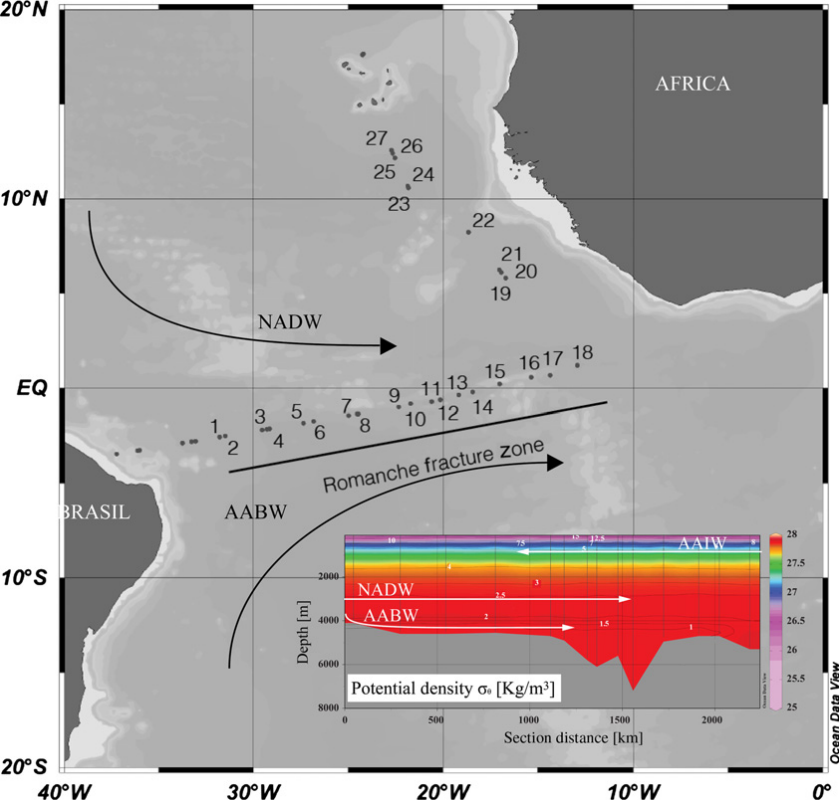
Stations occupied during ARCHIMEDES-3 in the (sub)tropical Atlantic. The Romanche Fracture Zone (RFZ) comprises Sts 1–18. The direction of flow of the major deep-water masses (Antarctic Intermediate Water AAIW, North Atlantic Deep Water NADW and Antarctic Bottom Water AABW) is shown in the inset. The inset shows the potential density (colored scale) and potential temperature (indicated by the numbers in °C and isothermals) and the direction and approximate depth of the core waters of the individual deep-water masses.

Water samples were taken at 18 stations (Sts 1–18) along a 2000-km-long transect through the Romanche Fracture Zone (RFZ), a deep but narrow canyon in the Mid-Atlantic Ridge at the equator (Fig. 1) and along a latitudinal transect from the equatorial region towards the north (St. 19–27; Fig. 1). Sampling was conducted during the ARCHIMEDES-III cruise (December 2007 to January 2008) onboard RV *Pelagia*. Water samples were collected with a Seabird CTD (conductivity-temperature-depth) rosette sampler equipped with 18 12-L Niskin bottles. The Niskin bottles were flushed with bleach and rinsed with ambient seawater by deploying them to 1000 m depth prior to the sampling campaign. Water was sampled from the lower euphotic zone (100 m depth), the South Atlantic Central Water (SACW), where the oxygen minimum zone (OMZ) is also located, the Antarctic Intermediate Water (AAIW), the upper, middle and lower North Atlantic Deep Water (uNADW, mNADW, lNADW) and the Antarctic Bottom Water (AABW). These water masses were distinguished based on their physical-chemical characteristics (Table 1). Samples were taken for prokaryotic abundance and diversity and heterotrophic prokaryotic activity as described below and for contextual parameters including viral diversity and production (De Corte *et al*., 10).

**Table 1:** Physical and chemical characteristics of the major water masses sampled along the Romanche Fracture Zone based on CTD profiles at the individual stations

Water mass	Depth (m)	Temperature (°C)	Salinity	Oxygen (μmol kg^−1^)	Nitrate (μmol kg^−1^)	Nitrite (μmol kg^−1^)	Ammonia (μmol kg^−1^)	Phosphate (μmol kg^−1^)	Silicate (μmol kg^−1^)
Subsurface	100	14.2–15.9	35.4–35.7	100.9–170.3	11.03–22.09	0.03–0.13	0.00–0.01	0.84–1.41	4.31–7.14
SACW OMZ	250	11.0–12.5	35.1–35.2	84.3–106.2	24.20–29.43	0.01–0.02	0.00–0.02	1.55–1.83	9.30–11.79
SACW	500	6.4–7.7	34.5–34.6	131.1–168.6	29.81–30.93	0.01–0.01	0.01–0.01	2.01–2.03	18.22–19.08
SACW average	250–500	6.4–12.5	34.5–35.2	84.3–168.6	24.20–30.93	0.01–0.02	bd–0.02	1.55–2.03	9.30–19.08
AAIW	750	4.7–5.1	34.4–34.5	145.5–165.1	33.20–34.42	bd–0.01	0.01–0.01	2.23–2.32	27.65–29.31
uNADW	1750	3.8–4.0	35.0	235.2–247.6	19.28–20.21	bd–0.01	0.00–0.01	1.27–1.33	16.21–17.71
mNADW	2750	2.4–2.8	34.9–35.0	238.1–246.6	20.76–21.71	bd–0.01	0.01–0.01	1.37–1.45	30.94–40.92
lNADW	3750–4000	2.1–2.4	34.9	245.2–254.2	19.56–21.42	bd–0.01	0.01–0.01	1.32–1.48	32.61–45.18
NADW average	1750–4000	2.1–3.9	34.9	235.2–254.2	19.28–21.71	bd–0.01	0.01–0.01	1.27–1.48	16.21–45.18
AABW	4500–7000	1.0–1.4	34.8	222.9–229.0	27.19–29.26	0.01–0.12	0.01–0.01	1.86–2.01	82.74–94.47

OMZ, oxygen minimum zone; SACW, South Atlantic Central Water; AAIW, Antarctic Intermediate Water; uNADW, upper North Atlantic Deep Water; mNADW, middle NADW; lNADW, lower NADW; AABW, Antarctic Bottom Water; bd, below detection limit.

### Prokaryotic abundance

The prokaryotic plankton were quantified by flow cytometry following Del Giorgio *et al*. (12) with modification. Briefly, water samples (2 mL) were fixed with 0.5% glutaraldehyde (final concentration), flash-frozen in liquid N_2_ for 5 min and stored at −80 °C until further analysis. Prior to analysis, the samples were thawed to room temperature and the prokaryotic cells stained with SYBRGreen I in the dark for 10 min. Subsequently, 1 μm fluorescent latex beads (approximately 1 × 10^5^ mL^−1^) (Molecular Probes, Invitrogen, Carlsbad, CA) were added to all the samples as internal standard. The prokaryotes were enumerated using a FACSCalibur flow cytometer (Becton Dickinson, Franklin Lakes, NJ) according to their signature in right angle light scatter and green fluorescence.

### Prokaryotic heterotrophic activity (PHA)

Bulk PHA was measured by adding 5 nmol L^−1^ [^3^H]-leucine (final concentration, specific activity 160 Ci mmol L^−1^, GE Healthcare, Amersham, Bucks, UK) to triplicate 10–40 mL samples and triplicate formaldehyde-killed blanks (2% final concentration) (Simon & Azam, 43). Samples and blanks were incubated in the dark at *in situ* temperature in temperature-controlled chambers for 4–24 h depending on the expected activity. Incubations were terminated by adding formaldehyde (2% final concentration) to the samples. After 10 min, the samples and the blanks were filtered onto 0.2-μm polycarbonate filters (25 mm filter diameter, Millipore) supported by HAWP filters (Millipore, 0.45 μm pore size). Subsequently, the filters were rinsed three times with 10 mL of 5% ice-cold trichloroacetic acid. Thereafter, the filters were transferred into scintillation vials and dried at room temperature. Scintillation cocktail (8 mL Filter Count, Perkin-Elmer, Waltham, MA) was added and after 18 h, the radioactivity was determined in a Tri-Carb 2910TR scintillation counter (Perkin-Elmer, Groningen, The Netherlands). The mean disintegrations per minute (DPM) of the formaldehyde-fixed blanks were subtracted from the mean DPM of the respective samples and the resulting DPM converted into leucine incorporation rates.

### DNA extraction of the prokaryotic community

Ten liters of seawater were filtered through a 0.22-μm Sterivex filter GP unit (Millipore). Subsequently, 1.8 mL of lysis buffer (40 mM EDTA, 50 mM Tris-HCl, 0.75 M sucrose) was added to the filters and stored at −80 °C until further processing in the laboratory. The DNA extraction was performed using Ultraclean Mega Soil DNA isolation Kit (MoBIO Laboratories, Carlsbad, CA) and the DNA extract was concentrated further (*c*. 10 times) using Centricon units (Millipore).

### PCR and T-RFLP analysis of bacterial and archaeal communities

PCR conditions and chemicals were applied as described by Moeseneder *et al*. (35). A 1-μL aliquot of the DNA extract was used as a template in a 50-μL PCR mixture. Bacterial 16S rRNA genes were amplified using the universal primers 27F-FAM and 1492R-JOE (Lane, 29). Archaeal 16S rRNA genes were amplified using the specific primers 21F-FAM and 958R-JOE (DeLong, 13). Amplification of the 16S rRNA started with a denaturation step at 94 °C (for 3 min), followed by 35 cycles of denaturation at 94 °C (1 min), annealing at 55 °C (1 min), and an extension at 72 °C (1 min). A final extension at 72 °C for 30 min completed the cycling. PCR products were run on a 1.0% agarose gel and subsequently stained with SYBRGold. The bands obtained were excised from the gel and purified with the Quick gel extraction kit (Genscript, Piscataway, NJ). The purified PCR product was quantified on a Nanodrop spectrophotometer. Digestion of the fluorescently labeled PCR products was done at 37 °C overnight. Each reaction contained 30 ng of cleaned PCR product, 5 U of tetrameric restriction enzyme (HhaI) and the respective buffer, filled up to a final volume of 50 μL with ultra-pure water (Sigma, St. Louis, MO). The restriction enzyme was heat-inactivated and the digested DNA was precipitated by adding 4.5 μL linear polyacrylamide (prepared with acrylamide, TEMED and APS in Tris-EDTA buffer) solution to 100 μL of 100% isopropanol. The samples were kept at room temperature for 15 min and subsequently centrifuged at 15 000 ***g*** for 15 min. Thereafter, the supernatant was discarded and the pellet rinsed with 100 μL of 70% isopropanol and precipitated again by centrifugation (15 000 ***g*** for 5 min). Subsequently, the supernatant was removed again and the DNA dried in the cycler at 94 °C for 1 min and stored at −20 °C until further analysis. The pellet was resuspended in 2 μL of ultra-pure water and the product denatured with 7.8 μL of Hi-Di formamide (highly deionized formamide for capillary electrophoresis) (Applied Biosystems, Foster City, CA) at 94 °C for 3 min. Each sample contained 0.2 μL of GeneTrace 1000 (ROX) marker (Applied Biosystems). Fluorescently labeled fragments were separated and detected with an ABI Prism 310 capillary sequencer (Applied Biosystems) run under genescan mode (Moeseneder *et al*., 34). The size of the fluorescently labeled fragment was determined by comparison with the internal GeneTrace 1000 (ROX) size standard. Injection was performed electrokinetically at 15 kV and 60 °C for 15 s (adjustable). The output from the ABI genescan software was transferred to the fingerprinting ii software (Bio-Rad) to determine the peak height and for standardization using the size marker. The obtained matrix was analyzed with primer software (Primer-E v. 6) to determine the similarity of the T-RFLP fingerprints between samples. The Relate function (primer-E v.6) was used to establish the correlation between the matrices of the prokaryotic community composition and the physical and chemical (salinity, temperature and nutrient concentrations) and biological parameters (prokaryotic abundance and leucine incorporation). Linear regression was used to inspect the relationship between the similarity in community composition and the distance between samples through the RFZ and at the eastern North Atlantic for different water masses. Since the data used for this analysis comprise pairwise comparisons implying lack of independence, bootstrapping (10 000 replications) was used to test whether the slope of the regression obtained for the different water masses and areas was different from zero (Efron & Tibshirani, 16; Horner-Devine *et al*., 25). Ancova was used to test whether the mean of the randomly generated slopes by bootstrapping was significantly different from the observed slope.

### Cloning, sequencing and phylogenetic analysis of *Bacteria* and *Archaea*

Cloning was done for *Bacteria* and *Archaea* at St. 12, located in the center of the RFZ. The number of bacterial clones sequenced from the 100 m depth horizon was 165, from the SACW 166, from the NADW 198 clones, and from the AABW 149 clones. The number of archaeal clones sequenced was 34 for the 100 m depth horizon, 32 for the SACW, 63 for the NADW and 55 for the AABW. The 16S rRNA genes of *Bacteria* were amplified with the universal primers 27F and 1492R (Lane, 29) and those of *Archaea* with the specific primers 21F and 958R (DeLong, 13). DNA 1 μL was used as template in a 50-μL PCR mixture containing 25 pmol of each primer, 200 μM of dNTPs, 1 U of Taq polymerase Biotherm (Genecraft), the corresponding buffer, and filled up to 50 μL with UV-treated ultra-pure water (Sigma). The amplification was performed by an initial denaturation step at 94 °C for 3 min, followed by 35 cycles of denaturation at 94 °C (1 min), annealing at 55 °C (1 min), and extension at 72 °C (1 min), and a final extension at 72 °C for 7 min. The quality of the PCR products was checked on a 2% agarose gel. The PCR product was cloned with the TOPO-TA cloning kit (Invitrogen) according to the manufacturer's instructions. Clones were checked for the right insert by running the PCR product on a 2% agarose gel. Sequencing was performed by Macrogen Inc. (Europe) using the 27F and 21F primer for *Bacteria* and *Archaea*, respectively. Sequences were 700 bp long and sequenced in one direction only. A similarity threshold of 98% was used to differentiate OTUs.

Sequence information obtained in this study has been deposited in GenBank: accession numbers JN710025 to JN710376 for *Bacteria* and accession numbers JN802140 to JN802178 for *Archaea*.

The sequence data were compiled using mega-4 software (Tamura *et al*., 47), and aligned together with environmental bacterial and archaeal sequences obtained from the NCBI database using rdp software (http://rdp.cme.msu.edu/). The sequences were checked for chimera using pintail software (Ashelford *et al*., 6). Phylogenetic analyses were conducted in mega-4. The evolutionary history was inferred using the neighbor-joining method and Jukes–Cantor distance matrix in mega-4 software and drawn using itol (Interactive Tree Of Life) (Letunic & Bork, 31). The phylogenetic affiliation of the bacterial and archaeal 16S rRNA genes was determined by naïve Bayesian classifier implemented in rdp (Wang *et al*., 52).

Rarefaction analysis was performed using mothur (Schloss *et al*., 42) for each sample and depth layer. OTUs were defined as a group of sequences differing by < 2%. The Chao index was also calculated using mothur. The phylogenetic composition of microbial communities was compared using unifrac (Lozupone & Knight, 32).

### CARD-FISH and FISH

CARD-FISH was used to determine the abundance of major groups of prokaryotes at six stations (Sts 1, 5, 9, 12, 15 and 17) in the RFZ following the method described by Pernthaler *et al*. (39). Immediately after collecting the samples from the Niskin bottles, 20–80 mL of water were fixed by adding 0.2 μm filtered paraformaldehyde (2% final concentration) and stored at 4 °C in the dark. After 12–18 h, the samples were filtered through 0.2-μm polycarbonate filters (Millipore GTTP, 25-mm filter diameter) supported by cellulose nitrate filters (Millipore, HAWP, 0.45 μm), washed twice with 10 mL Milli-Q water, dried and stored in a microfuge vial at −20 °C until further processing in the laboratory. For hybridization, we used horseradish peroxidase-labeled oligonucleotide probes to determine the fraction of the following groups on the abundance of 4′,6-diamidino-2-phenylindole (DAPI)-stained cells *Thaumarchaeota*, *Bacteria*, SAR11 (*Alphaproteobacteria*), SAR324 (*Deltaproteobacteria*), SAR406, and *Alteromonas* (*Gammaproteobacteria*). The probes and hybridization conditions for the individual groups are given in Supporting Information Table S1. The tyramide signal amplification was performed according to Teira *et al*. (48). The SAR324 hybridization and tyramide signal amplification was done following Schattenhöfer *et al*. (41).

FISH with Cy3-labeled probes was used to enumerate the members of the SAR202 (*Chloroflexi*) cluster. The hybridization conditions for the SAR202 cluster were applied as described by Morris *et al*. (37) (Table S1). Cells were counter-stained with a DAPI-mix [5.5 parts of Citifluor (Citifluor), 1 part of Vectashield (Vector Laboratories) and 0.5 parts of phosphate-buffered saline (PBS) with DAPI (final concentration 2 μg mL^−1^)]. Enumeration of DAPI-stained cells and cells stained with the specific probes was performed under a Zeiss Axioplan 2 epifluorescence microscope equipped with a 100-W Hg lamp and appropriate filter sets for DAPI, Cy3 and Alexa448. A minimum of 600 DAPI-stained cells was counted per sample.

## Results

### Water mass characteristics

**Figure 2:**
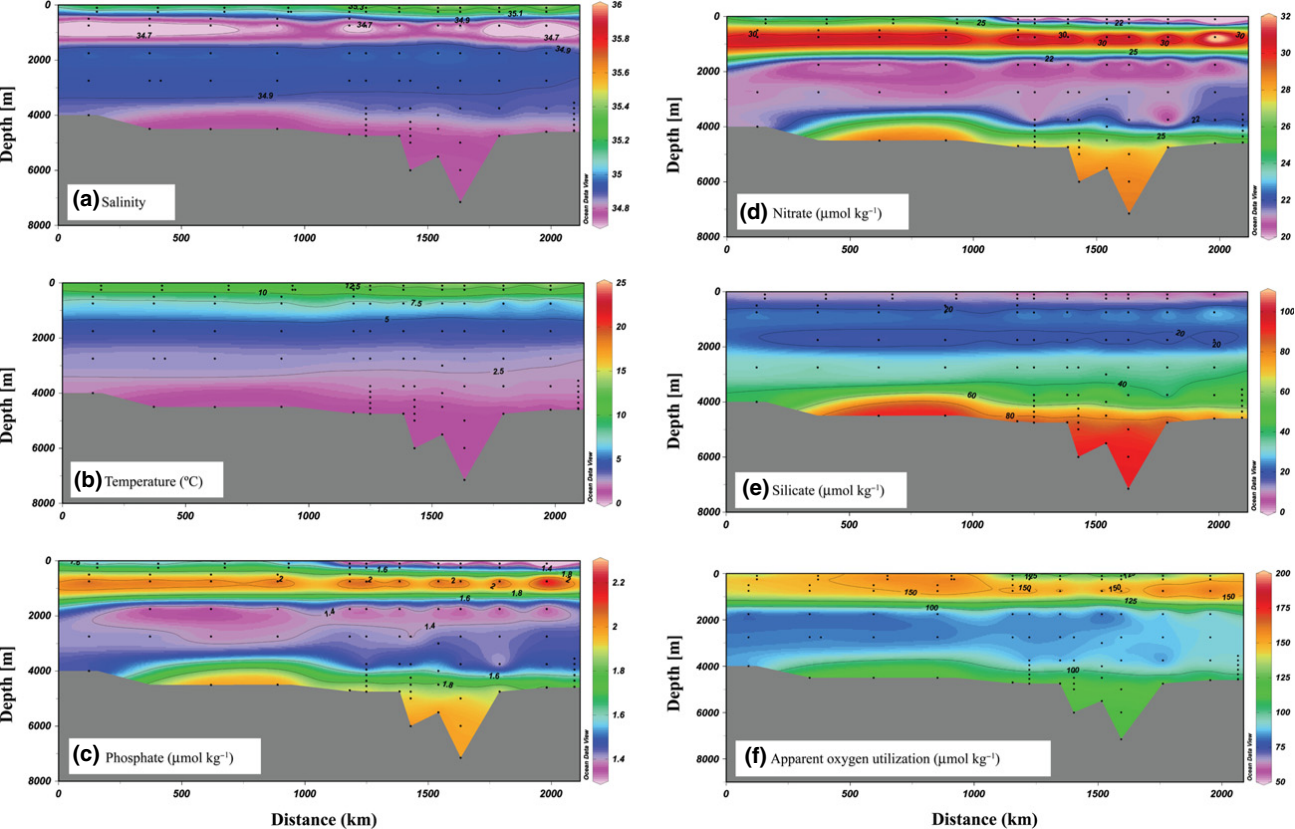
Distribution of salinity (a), temperature (b), phosphate (c), nitrate (d), silicate (e) and apparent oxygen utilization (f) along the Romanche Fracture Zone (Sts 1–18). Sampling depths and locations are indicated by dots.

The basic physical and chemical characteristics of the main water masses are summarized in Table 1. The patterns of salinity, temperature, inorganic nutrients (phosphate, nitrate and silicate) and apparent oxygen utilization (AOU) in the RFZ are shown in Fig. 2. The AABW was found below 4000 m depth, characterized by low salinity (34.8), low temperature (1.0–1.4 °C) and high silicate concentrations (82.74–94.47 μmol kg^−1^). The NADW was clearly identifiable between 1750 and 4000 m depth by its characteristic salinity maximum (34.9–35.0). Three layers of the NADW were distinguished: the upper NADW (uNADW) with a temperature of 3.8–4.0 °C, the middle NADW (mNADW) with high concentrations of phosphate (1.37–1.45 μmol kg^−1^) and nitrate (20.76–21.71 μmol kg^−1^), and the lower NADW (lNADW) with low temperatures (2.1–2.4 °C) and high oxygen concentrations (245.23–254.24 μmol kg^−1^). The AAIW was detected at around 750 m depth, characterized by low salinity (34.4–34.5), high phosphate (2.23–2.32 μmol kg^−1^) and nitrate (33.20–34.42 μmol kg^−1^) concentrations (Fig. 2). The South Atlantic Central Water (SACW) with its OMZ was located at around 250 m depth with oxygen concentrations of 84.3–106.2 μmol kg^−1^ and an AOU of *c*. 150 μmol kg^−1^ (Fig. 2f).

### Prokaryotic abundance and leucine incorporation

**Figure 3:**
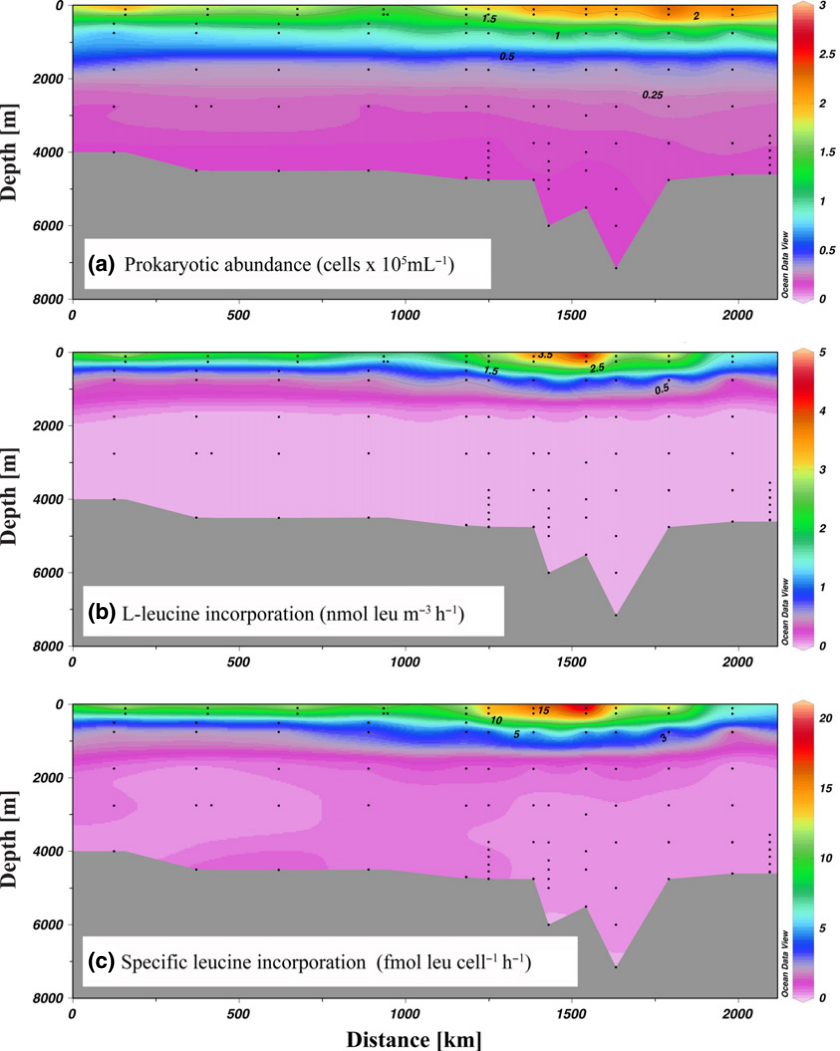
Distribution of prokaryotic abundance (a), prokaryotic heterotrophic production (b) and cell-specific leucine incorporation (c) along the Romanche Fracture Zone (Sts 1–18). Sampling depths and locations are indicated by dots.

The highest prokaryotic abundance was detected in the subsurface (100 m depth horizon) at the eastern side of the RFZ, declining exponentially with depth. In the AABW below 6000 m depth, mean prokaryotic abundance was 1.7 ± 2.3 × 10^4^ cells mL^−1^ (Fig. 3a). Leucine incorporation followed a similar spatial pattern as prokaryotic abundance, decreasing from the subsurface (3.8 ± 4.6 nmol Leu m^−3^ h^−1^) by three orders of magnitude in the layers below 6000 m depth (3.4 ± 2.6 × 10^−3^ nmol Leu m^−3^ h^−1^ (Fig. 3b). Cell-specific leucine incorporation ranged from 16.8 ± 6.9 fmol Leu cell^−1^ h^−1^ in the subsurface layer to 0.32 ± 0.15 fmol Leu cell^−1^ h^−1^ in the AABW below 6000 m depth (Fig. 3c).

### Bacterial and archaeal community composition determined by T-RFLP fingerprinting

**Figure 4:**
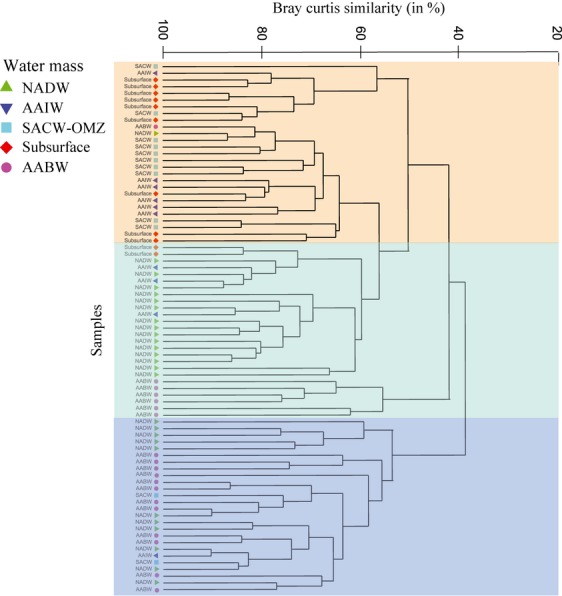
Clustering of samples from different depths based on Bray–Curtis similarity matrix obtained by T-RFLP fingerprinting of bacterial communities. Different symbols denote water masses. For water mass abbreviations see Table 1.

The T-RFLP pattern of the bacterial community revealed a total of 116 OTUs, with fragments ranging from 34 to 966 bp (data not shown). Forty-four OTUs were found in the subsurface, 71 OTUs in the intermediate waters, 47 OTUs in the NADW and 62 OTUs in the AABW. Twenty-one (or 18%) of the 116 OTUs were present in all the water masses. In all, 7% of the OTUs were present in more than 70% of the samples and 25 OTUs (or 22%) were unique to specific water masses. These 22% of OTUs specific to distinct water masses led to a clear separation of bacterial communities according to the major water masses with three main clusters (Fig. 4). One cluster comprised the bacterial communities of subsurface and the SACW (labeled in orange in Fig. 4), another cluster consisted mainly of the bacterial communities of the NADW (in green, Fig. 4), and the third cluster comprised mostly the bacterial communities of the AABW (in blue, Fig. 4).

**Figure 5:**
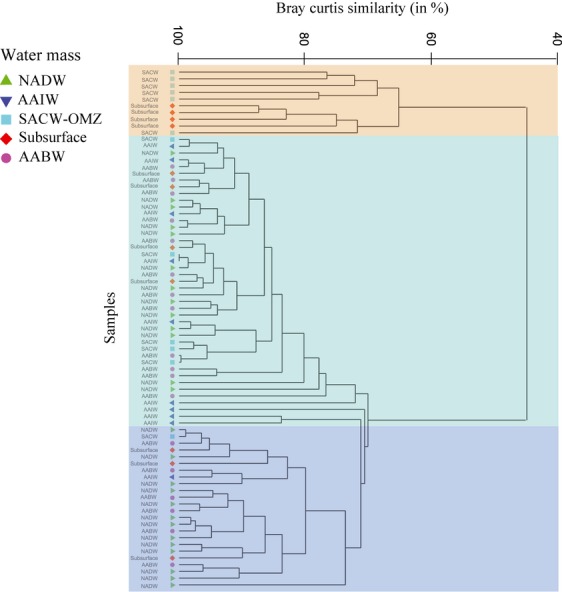
Clustering of samples from different depths based on Bray–Curtis similarity matrix obtained by T-RFLP fingerprinting of archaeal communities. Different symbols denote water masses. For water mass abbreviations see Table 1.

Within the archaeal community, 25 OTUs were detected with fragments ranging from 71 to 919 bp (data not shown). Two OTUs (324 bp, 333 bp) were present in all samples. Nine archaeal OTUs were found in the subsurface layer, 15 in intermediate waters, 10 in NADW and 6 in AABW. One unique OTU was present in the subsurface, 4 OTUs in the AAIW and one in the NADW. Three major clusters of the archaeal communities were identified, one composed of the subsurface waters and the SACW, the second one composed mainly of AAIW, NADW and AABW, and the third cluster composed mainly of AABW and NADW (Fig. 5).

The oxygen minimum zone (OMZ) of the SACW exhibited the highest bacterial and archaeal diversity, with Shannon diversity indices (H') of 2.69 and 1.22 for *Bacteria* and *Archaea*, respectively (Table 2). Margalef's index (SR) was 5.98 for *Bacteria* and 0.89 for *Archaea* (Table 2). In contrast to the SACW, the NADW exhibited the lowest bacterial diversity, with H' = 2.29 and SR = 4.02 (Table 2). *Archaea* also exhibited a lower diversity in the deep-water masses than in the upper waters, with H' = 0.80 and SR = 0.51 for NADW, and H' = 0.83 and SR = 0.48 for AABW (Table 2).

**Table 2:** Margalef's species richness (SR), Shannon index (H′) obtained from 16S rRNA gene clone libraries and T-RFLP. For water mass abbreviations see Table 1

Water mass	Samples	Technique	SR	H′
Subsurface	*Bacteria*	TRFLP	5.00	2.64
SACW OMZ	*Bacteria*	TRFLP	5.98	2.69
AAIW	*Bacteria*	TRFLP	4.50	2.66
NADW	*Bacteria*	TRFLP	4.02	2.29
AABW	*Bacteria*	TRFLP	4.10	2.38
Subsurface	*Archaea*	TRFLP	0.60	1.03
SACW OMZ	*Archaea*	TRFLP	0.89	1.22
AAIW	*Archaea*	TRFLP	0.54	0.90
NADW	*Archaea*	TRFLP	0.51	0.80
AABW	*Archaea*	TRFLP	0.48	0.83
Subsurface	*Bacteria*	Cloning	22.13	4.53
SACW OMZ	*Bacteria*	Cloning	21.71	4.37
NADW	*Bacteria*	Cloning	18.72	3.85
AABW	*Bacteria*	Cloning	18.23	4.23
Subsurface	*Archaea*	Cloning	5.38	2.83
SACW OMZ	*Archaea*	Cloning	3.17	2.11
NADW	*Archaea*	Cloning	2.45	1.94
AABW	*Archaea*	Cloning	2.25	1.69

OMZ, oxygen minimum zone.

The physical and chemical characteristics of the different water layers were compared with the matrix of the microbiological factors using the Relate function (a comparative Mantel-type test) of the primer software (Table 3). A significant correlation was obtained between the physical, chemical and microbiological characteristics (prokaryotic abundance, prokaryotic leucine incorporation and cell-specific activity) of the water masses (*R* = 0.793, *P* < 0.001). Also, the bacterial and archaeal community composition obtained by T-RFLP was related significantly to the physical and chemical parameters (Table 3). However, the bacterial community composition was more closely related to the physico-chemical factors (*R* = 0.396) than was the archaeal community composition (*R* = 0.215) (Supporting Information Fig. S1, Table 3).

**Table 3:** Relation between specific factors and bacterial and archaeal community composition using RELATE analysis (using primer-E v.6 software)

Comparison	*R*	*P*
Physico-chemical factors vs. biological factors	0.793	0.001
Physico-chemical factors vs. bacterial composition	0.396	0.001
Biological factors vs. bacterial CC	0.287	0.001
Physico-chemical factors vs. archaeal CC	0.215	0.001
Biological factors vs. archaeal CC	0.106	0.001
Bacterial composition vs. archaeal CC	0.106	0.005

CC, community composition.

### Bacterial and archaeal diversity derived from clone libraries

Rarefaction analysis revealed that our sequencing effort was not sufficient to recover the majority of the *Bacteria*. The Chao richness indices calculated for the bacterial community in the different water masses ranged from 150 to 290 OTUs (Fig. S2a) and for the archaeal community the Chao index was < 20 OTUs for all the individual water masses except for the 100 m layer (46 OTUs) (Fig. S2b). Therefore, based on the Chao index, we missed at least 50% of the bacterial OTUs present. The majority of the archaeal community, however, was covered by our sequencing effort, except for the subsurface waters, where we recovered only about 50% of the archaeal OTUs predicted by the Chao index (Fig. S2b).

The species-rank distribution of bacterial OTUs determined by clone libraries as well as from T-RFLP fingerprints indicates that only a few bacterial OTUs predominate in deeper water masses, whereas in subsurface and mesopelagic waters, the bacterial community is more evenly distributed (Fig. S3a and c). In the NADW, *Gammaproteobacteria* were highly diverse, particularly the genus *Alteromonas*, constituting 63% of the clone library retrieved from this water mass. The species-rank distribution of *Archaea* obtained by cloning and T-RFLP was generally similar to that of *Bacteria*, with a lower evenness in the deep waters (NADW, AAWB) (Fig. S3b and d).

**Figure 6:**
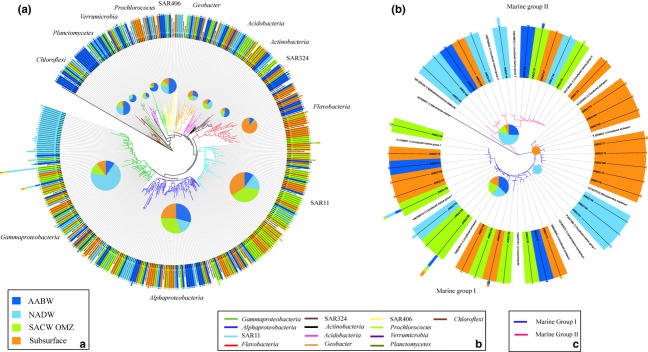
Neighbor-joining phylogenetic tree based on 16S rRNA gene analyses of *Bacteria* (a) and *Archaea* (b) obtained at a central station (St. 12) in the Romanche Fracture Zone. Only one representative of OTU sharing ≥ 98% identity is shown. Bars show the number of clones represented by each sequence. Four water masses are indicated by color code (a) and the proportions of clones from each depth represented in the different clusters are indicated in the pie charts. Phyla are distinguished by color of the branch in the phylogenetic tree of the *Bacteria* (see b) and *Archaea* (see c).

The phylogenetic composition of *Bacteria* and *Archaea* changed significantly with depth as revealed by the unifrac significance test (*P* < 0.01), although about 22% of the bacterial and 24% of the archaeal OTUs were ubiquitously present. The phylogenetic affiliation of *Bacteria* and *Archaea* is shown in Fig. 6. The main bacterial groups retrieved were *Gammaproteobacteria* (47%), *Alphaproteobacteria* (29%) and *Bacteroidetes* (9%). *Alteromonas* was the most abundant genus of the *Gammaproteobacteria* accounting for 90% of all the *Gammaproteobacteria* in the NADW (Fig. 6A). The ubiquitously present SAR11 group of the *Alphaproteobacteria* comprised sequences originating mainly from the OMZ of the SACW (38%) and the subsurface (34%). The *Flavobacteria* cluster within the *Bacteroidetes* consisted mainly of clones from the subsurface waters (86%).

The archaeal community comprised members of the *Thaumarchaeota* and Marine Group II *Euryarchaeota*. A high proportion of the clones belonging to the marine Group II *Euryarchaeota* (54%) originated from the NADW (Fig. 6B). Marine *Thaumarchaeota* exhibited two subclusters: one subcluster was closely related to *Nitrosopumilus maritimus* and contained clones from all the water masses, whereas the other subcluster contained only sequences from the subsurface layer (Fig. 6B).

The diversity indices calculated based on the clone libraries were generally higher than those based on T-RFLP analysis (Table 2). For *Bacteria*, the highest Shannon index was obtained for the subsurface waters (H′ = 4.53) and the lowest for the NADW (Table 2), coinciding with the large proportion of clones from the genus *Alteromonas* (62.5% of all the clones from NADW) found there. Similar to *Bacteria*, the highest Shannon index for *Archaea* was obtained for the subsurface waters (Table 2). The lowest Shannon index for *Archaea*, however, was not obtained in the NADW, as for *Bacteria*, but in the AABW (Table 2).

Principal component analyses of the phylogenetic composition of the bacterial and archaeal community of the different water masses (with components P1 and P2 explaining 70% of the variation) revealed high similarities between the NADW and AABW for both the bacterial and archaeal community composition retrieved by clone libraries (Fig. S4).

### Prokaryotic community composition determined by CARD-FISH

Using CARD-FISH to determine the abundance of specific phylogenetic groups of *Bacteria* resulted in a coverage of 41–107% of the *Bacteria* detected with all the probes applied in this study (Table 4). Thus, it appears that with the five bacterial oligonucleotide probe mixtures used, the major fraction of the bacterial community was detected.

**Table 4:** Average ± SD (*n* = 6, except for AABW where *n* = 5) of the contribution of *Bacteria* and *Thaumarchaeota* to the total prokaryotic community and of specific bacterial groups to the total bacterial abundance determined by FISH or CARD-FISH. For water mass abbreviations see footnotes to Table 1

Water mass	*Bacteria*	*Thaumarchaeota*	SAR11	SAR202	*Alteromonas*	SAR406	SAR324	Σ% bacterial groups
Subsurface	61.5 ± 3.1	16.2 ± 5.4	13.5 ± 6.1	5.3 ± 1.4	14.3 ± 2.8	4.5 ± 3.1	4.7 ± 1.1	42.3 ± 14.5
SACW	64.4 ± 6.1	17.0 ± 6.6	10.7 ± 3.9	4.3 ± 3.6	13.9 ± 2.5	4.5 ± 3.3	7.3 ± 3.8	40.7 ± 17.1
AAIW	61.5 ± 3.1	23.3 ± 7.6	16.1 ± 7.95	16.2 ± 7.1	18.5 ± 6.8	12.6 ± 6.5	16.6 ± 7.6	80 ± 35.9
NADW	67.1 ± 4.6	20.0 ± 4.8	11.4 ± 2.1	24.9 ± 8.9	17.8 ± 3.5	18.4 ± 3.8	23.5 ± 7.5	96 ± 25.8
AABW	68.4 ± 4.9	31.2 ± 3.9	9.9 ± 3.4	34.4 ± 12.2	20.3 ± 2.9	21.1 ± 7.5	21.9 ± 7.3	107.6 ± 33.3

**Figure 7:**
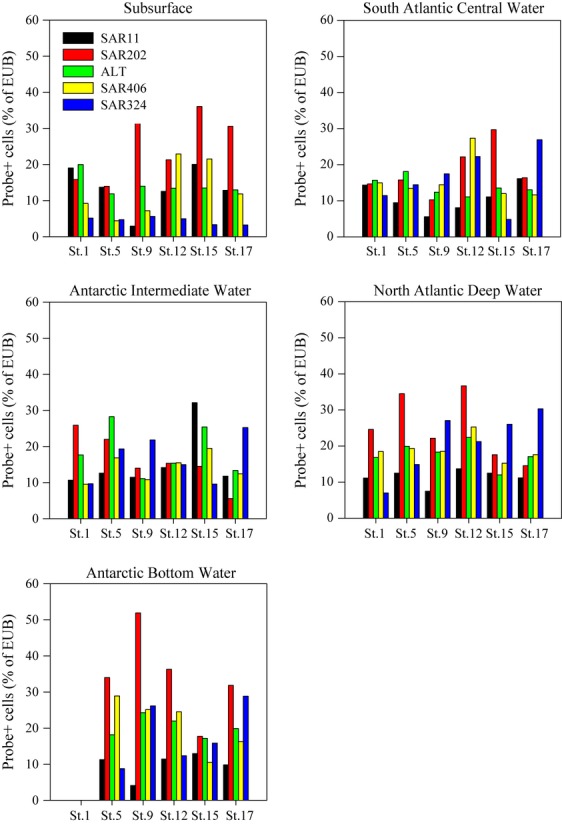
Contribution of specific bacterial groups (SAR11, SAR202, SAR324, SAR406, *Alteromonas*) to total bacterial abundance (Eub + cells) determined by FISH and CARD-FISH at selected stations in the different water masses (Subsurface, South Atlantic Central Water, Antarctic Intermediate Water, North Atlantic Deep Water and Antarctic Bottom Water) in the Romanche Fracture Zone.

The contribution of *Bacteria* to total prokaryotic abundance significantly increased from the subsurface waters (62%) to AABW (68%, *t*-test, *P* < 0.05) with no significant (*t*-test, *P* = 0.42) longitudinal trend through the RFZ. The contribution of *Thaumarchaeota* to total prokaryotic abundance in the subsurface waters (16%) was about half of that in the AABW (31%, *t*-test, *P* < 0.01) (Table 4). The contribution of *Thaumarchaeota* to total prokaryotic abundance was lower (Kruskal–Wallis one-way anova, *P* = 0.05) at the westernmost station (St. 1) than at the other stations, ranging from 8% at the subsurface layer to 14% in the NADW. The specific groups of *Bacteria* showed distinct distribution patterns along the transect (Fig. 7). Whereas SAR11 (*P* = 0.199) did not change, SAR202 (*P* < 0.001), SAR324 (*P* < 0.001), SAR406 (*P* < 0.001) and *Alteromonas* (*P* = 0.019) increased in their contribution to total bacterial abundance with depth (Table 4).

### Comparing community composition obtained by CARD-FISH and clone libraries

The contribution of members of the clades SAR202 and SAR406 to the bacterial community as determined by CARD-FISH and clone libraries was close to the 1:1 line, except for the subsurface layer, indicating that both techniques retrieved these clusters with similar efficiency (Fig. S5). In contrast, the relative abundance of the SAR11 cluster was higher (paired *t*-test, *P* < 0.05) in the clone libraries than with CARD-FISH. *Alteromonas* contributed disproportionally more to bacterial abundance using CARD-FISH than to *Bacteria* in clone libraries, with one outlier in the NADW (Fig. S5). The contribution of the SAR324 clade to bacterial abundance using CARD-FISH and clone libraries was generally variable (Fig. S5).

### Successional changes in bacterial community composition of specific water masses along the RFZ

**Figure 8:**
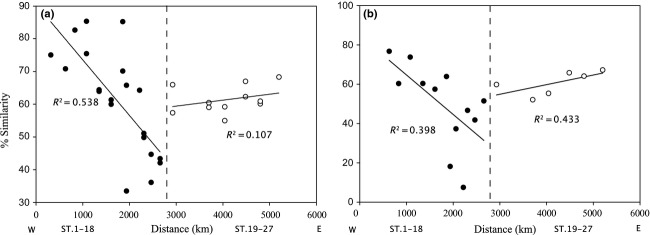
Successional change in (a) the bacterial and (b) the archaeal community composition of the NADW through the RFZ (black dots, Sts 1–18) and towards the north in the eastern Atlantic basin (open dots, Sts 19–27) based on T-RFLP analyses. The eastern exit of the NADW from the RFZ is indicated by the vertical dashed line. Percent of similarity is given in relation to the community composition of St. 1.

As the individual deep-water masses are funneled through the RFZ in a rather confined way over a period of *c*. 4 months for NADW and *c*. 7 months for AABW, we tested whether successional changes in the bacterial community composition, as determined by T-RFLP, are detectable. For the AAIW and AABW no significant changes in the bacterial community composition (AAIW *R*^2^ = 0.062 and AABW *R*^2^ = 0.036) were detectable along their flow through the RFZ. However, in the NADW the bacterial community composition gradually changed along its eastward path through the RFZ (Fig. 8a, *R*^2^ = 0.538, *P* < 0.001). The bacterial community of the eastern branch of the NADW (sampled at Sts 19–27) flowing along the eastern slope of the Mid-Atlantic Ridge towards the Atlantic sector of the Southern Ocean was more similar (between 55% and 70%) to the bacterial community of the NADW at the western entrance of the RFZ than to that of the eastern exit (35–40%) (Fig. 8a); however, it did not change significantly with distance (*R*^2^ = 0.107, *P* > 0.3). The qualitative changes in the bacterial community composition in the NADW flowing through the RFZ were accompanied by quantitative changes in some major bacterial clusters as well. The contribution of SAR324 to the total bacterial abundance increased (*t*-test, *P* < 0.05) from west to east (from 12% to 26%) through the RFZ (Fig. 7). In contrast, the fraction of the total bacterial community identified as SAR202 decreased from 25% in the NADW at the western entrance of the RFZ to 15% at the eastern exit (*t*-test, *P* = 0.12) (Fig. 7).

Similar to the successional change in the bacterial community, the archaeal community also changed in the NADW of the RFZ (Fig. 8B, *R*^2^ = 0.398, *P* < 0.03) but did not significantly change in the eastern Atlantic basin (*R*^2^ = 0.433, *P* > 0.1). The observed slopes were not significantly different from the randomly generated slopes (*P* > 0.9) for either *Bacteria* or *Archaea*.

## Discussion

The overarching hypothesis of this paper is that differences in the origin and history of the deep-water masses do not only lead to distinct salinity-temperature and nutrient characteristics but are also reflected in differences in the prokaryotic community composition and activity between individual deep-water masses. Fracture zones in the Mid-Atlantic Ridge with their characteristic bidirectional flow of the deep-water masses facilitate estimates of successional changes of prokaryotic communities potentially occurring during the passage of these deep-water masses through these fracture zones. In this study, we focused on the RFZ as a major conduit of NADW and AABW from the western into the eastern basin of the Atlantic.

### Methodological considerations

Differences between the relative contribution of specific bacterial groups to *Bacteria* using clone libraries and FISH, such as the higher contribution of SAR11 to clone libraries (this study; Cottrell & Kirchman, 9; Morris *et al*., 36; Alonso-Sáez *et al*., 4; De Corte *et al*., 11), might be caused by mismatches of the FISH probes or by the amplification efficiency in the PCR. To determine the coverage of our oligonucleotide probes, we matched them against the clone libraries using geneious software. The coverage of the probes used to enumerate members of the SAR11 and SAR202 cluster was 100% for the respective clones and 72% for SAR406. This indicates that for SAR11 and SAR202, probe mismatch is not a likely cause of the discrepancies between the estimated contribution using FISH and clone libraries. However, due to the limited length of our sequences, not all the probes were suited for this assessment. The specificity of the probe used to determine SAR324 was analyzed with sequences from the open ocean retrieved by Illumina from a previous study (unpublished data) revealing specificity for a cluster within the SAR324 (28 within group hits and seven non-group hits).

### Water-mass specific community composition of prokaryotes

Analyses of the T-RFLP fingerprints and of the clone libraries, indicate stratification of the bacterial and, to a lesser extent, the archaeal communities, with three major clusters associated with distinct water masses. Consequently, bacterial and archaeal community structure were related (*P* < 0.001) to biological and environmental factors similar to abundance and activity (Table 3). Agogué *et al*. (1), investigating the bacterial community composition of the major water masses in the North Atlantic, suggested that the water mass-specificity of bacterial communities is due to a few abundant OTUs and, more importantly, by less abundant OTUs, which constitute the ‘rare biosphere’ (Sogin *et al*., 45). Although the T-RFLP approach used in this study has a much lower resolution than the pyrosequencing approach used in Agogué *et al*. (1), we were nonetheless able to resolve this water mass specificity of the bacterial and archaeal communities. Similar to the findings of Agogué *et al*. (1), the highest number of OTUs was found for *Bacteria* in the NADW as determined by rarefaction analysis of the clone libraries. Archaeal richness, however, was highest in the subsurface layer, decreasing with depth. CARD-FISH results also support this stratification of the bacterial and archaeal communities.

The abundance of the SAR11 cluster of the *Alphaproteobacteria* subclass*,* considered to be the most abundant bacterial group in the ocean (Giovannoni & Rappé, 22; Morris *et al*., 36), did not decrease with depth, in agreement with a previous study using pyrosequencing in North Atlantic waters (Agogué *et al*., 1). Conversely, the contributions of SAR202, SAR324, SAR406 and *Alteromonas* to total bacterial abundance increased with depth, confirming previous reports from the Atlantic (Varela *et al*., 51; Agogué *et al*., 1). Fuchs *et al*. (18) did not detect SAR406 below the chlorophyll maximum layer in the Arabian Sea, in contrast to our findings. Agogué *et al*. (1) report that SAR406 increases in relative abundance with depth. The deltaproteobacterial clade SAR324 exhibited the highest relative abundance in the NADW and AABW (Table 4). Swan *et al*. (46), using a single-cell genomics approach, showed that members of the SAR324 clade are chemoautotrophs harboring RuBisCO and sulfur-oxidizing genes in the meso- and bathypelagic water column. The contribution of *Thaumarchaeota* to total prokaryotic abundance also increased with depth, as previously reported for the Atlantic (Varela *et al*., 50). Members of this group have been related frequently to chemoautotrophy harboring the genes encoding ammonia monooxygenase, responsible for ammonia oxidation (Herndl *et al*., 24; Könneke *et al*., 28; Hallam *et al*., 23). In the deep waters of the RFZ, the ratio of archaeal *amo*A to 16S rRNA gene of *Thaumarchaeota* is close to 1 (Sintes *et al*., 44). The increase in relative abundance of SAR324 and *Thaumarchaeota* with depth supports the notion that chemolithoautotrophy in the dark ocean's water column might be more important than commonly assumed (Reinthaler *et al*., 40; Swan *et al*., 46).

The increase in relative abundance of *Alteromonas* with depth reflects the general increase of *Gammaproteobacteria* with depth as reported elsewhere (DeLong *et al*., 14; Lauro *et al*., 30). Most of what is known from *Alteromonas* in the deep ocean is based on the ‘deep-water strain’ of *Alteromonas macleodii* which has been characterized as an r-strategist with a large genome size, frequently associated with particles (Garcia-Martinez *et al*., 21). This ‘deep-water strain’ of *A. macleodii* shows adaptations to high-pressure conditions and to the degradation of recalcitrant organic matter (Ivars-Martínez *et al*., 26).

### Successional changes in the prokaryotic community composition through the RFZ

The RFZ, where the NADW and AABW flow from west to east and the AAIW from east to west along a rather constrained path, allows the determination of the successional changes in the prokaryotic community over a period of 4–7 months of water mass transport through the RFZ (recalculated from Mercier & Speer, 33). The bacterial and archaeal community composition of the NADW gradually changes along the flow path and becomes increasingly dissimilar to the community composition in the NADW at the western entrance of the RFZ.

Whereas successional changes in the NADW flowing through the RFZ are apparent for both *Bacteria* and *Archaea*, no successional changes in the archaeal and bacterial community composition were detectable in the AABW and AAIW along their flow through the RFZ. The reason for the lack of successional changes in the AABW might be the low but continuous supply of organic and/or inorganic nutrients from the slope and bottom sediments, and a generally relatively high abundance of particles in AABW throughout the RFZ (Bochdansky *et al*., 7). Although there might be some input of nutrients to AABW from below due to diffusion out of the sediment, the AAIW likely receives input from the overlying waters through sinking particulate organic carbon (POC). In the RFZ, input of POC from surface waters might be fueled by the equatorial upwelling and the accompanied higher primary production. Hence, the successional changes in the bacterial and archaeal communities in the NADW are likely caused by the lack of input of additional nutrients leading to gradual changes in the available source of energy and, consequently, to changes in the prokaryotic community composition over the transit period of NADW in the RFZ of about 4 months. These deep-water successional changes are provoked most probably by subtle changes in nutrient availability and not by grazing impact, as deep-water prokaryotic communities are generally considered to be resource-limited and not controlled by grazers or viral lysis, despite the high virus prokaryote ratio in deep waters (Parada *et al*., 38; Arístegui *et al*., 5; De Corte *et al*., 10).

Taken together, we have shown that bacterial and archaeal communities undergo successional changes in the NADW over a period of *c*. 4 months in a similar way as surface water prokaryotes over seasonal cycles. These successional changes within distinct deep water masses in the absence of input of nutrients indicate that deep-water prokaryotic communities are adapting to subtle physical and biogeochemical changes in the water masses within a time frame of weeks to months.

## Supporting Information

**Figure S1.** Multidimensional scaling plots: physicochemical factors (A), biological factors (B), bacterial community composition (C) and archaeal community composition (D).

**Figure S2.** Rarefaction analysis of the clone libraries of the bacterial (A) and archaeal (B) communities obtained from the different water masses at St. 12. The Chao index for the OTUs sharing 98% identity is indicated for each water mass by horizontal lines.

**Figure S3.** Rank distribution of bacterial OTUs assessed by cloning and sequencing (A) and T-RFLP fingerprinting (C) and of archaeal OTUs assessed by cloning and sequencing (B) and T-RFLP fingerprinting (D).

**Figure S4.** Principal Component analysis of the bacterial (A) and archaeal (B) communities obtained along the Romanche Fracture Zone.

**Figure S5.** Relative contribution of specific bacterial groups to the bulk bacterial abundance determined by FISH and CARD-FISH vs. the percentage of cells of the respective bacterial group retrieved by 16S rRNA gene clone libraries.

**Table S1.** Overview of the probes and hybridization conditions applied in (CARD-)FISH analysis.
