# Supplementary figures and images for: Spatial patterns of bacterial and archaeal communities along the Romanche Fracture Zone (tropical Atlantic)
Source: FEMS Microbiol Ecol. 2013 May 16;85(3):537–52. doi: 10.1111/1574-6941.12142 (PMC3840699; doi:10.1111/1574-6941.12142)

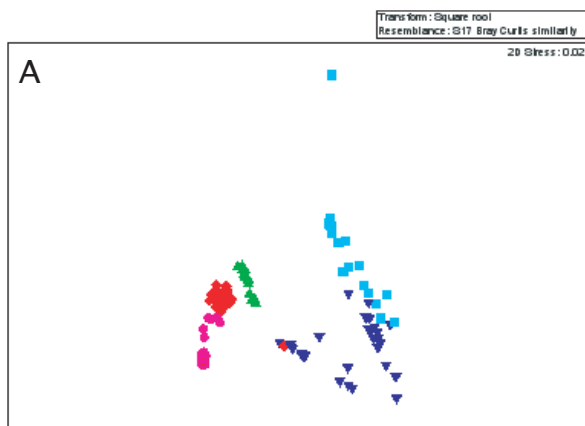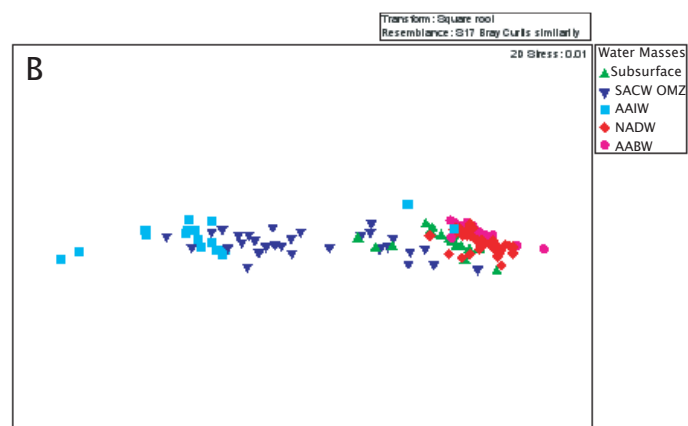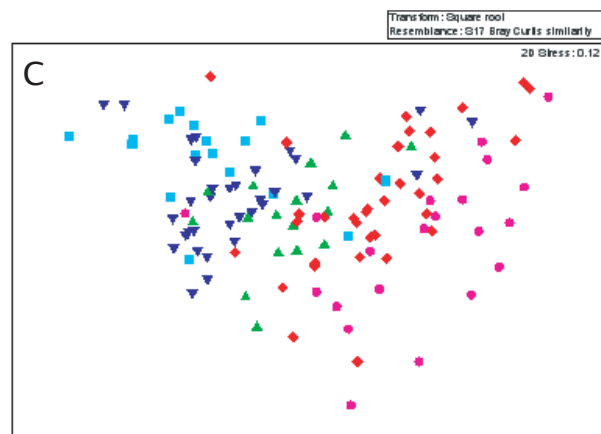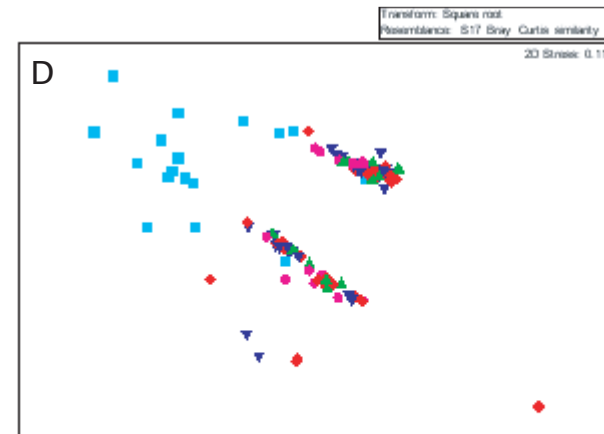

Sup.Fig.1

Supplement: Supplementary file 1 — Figure S1. Multidimensional scaling plots: physicochemical factors (A), biological factors (B), bacterial community composition (C) and archaeal community composition (D). [file fem0085-0537-sd1.pdf]

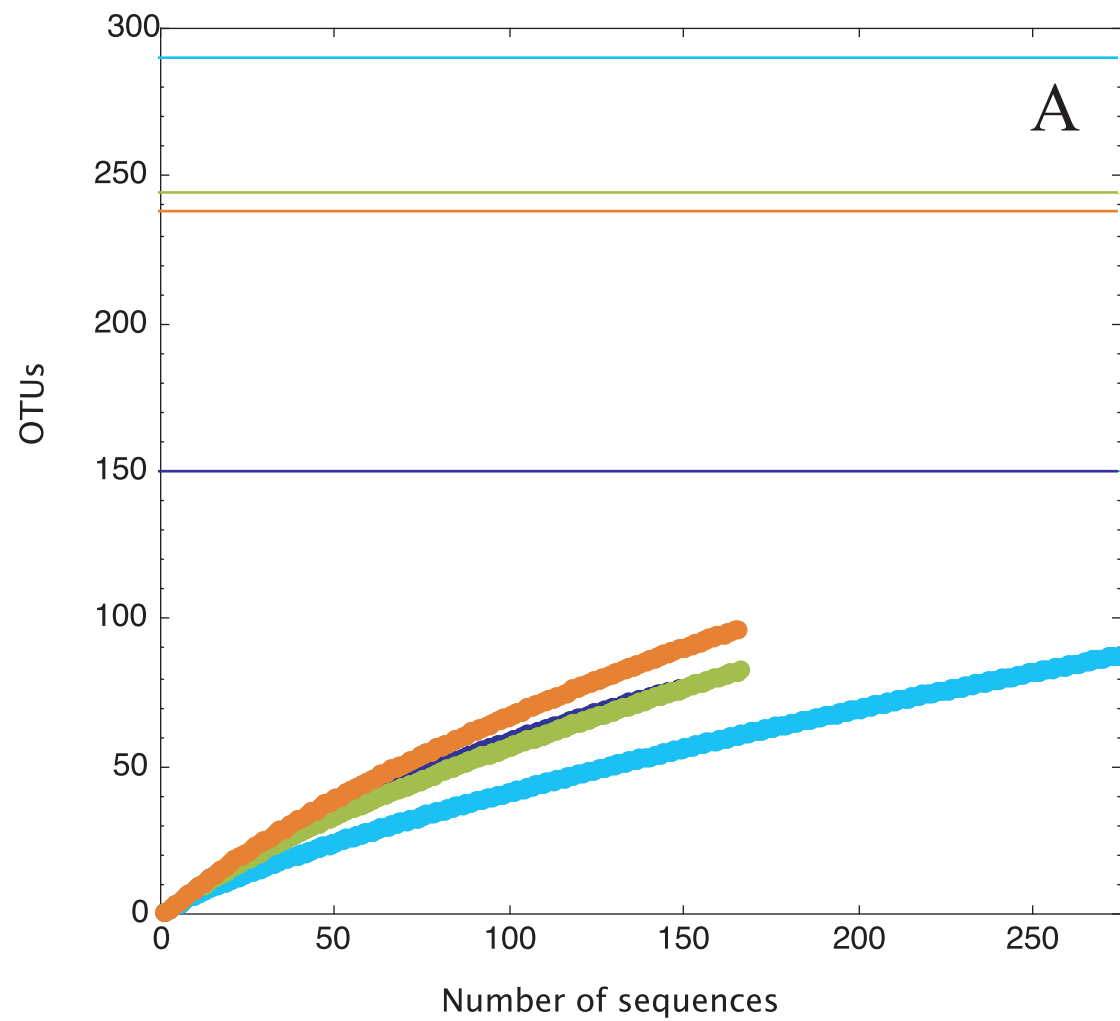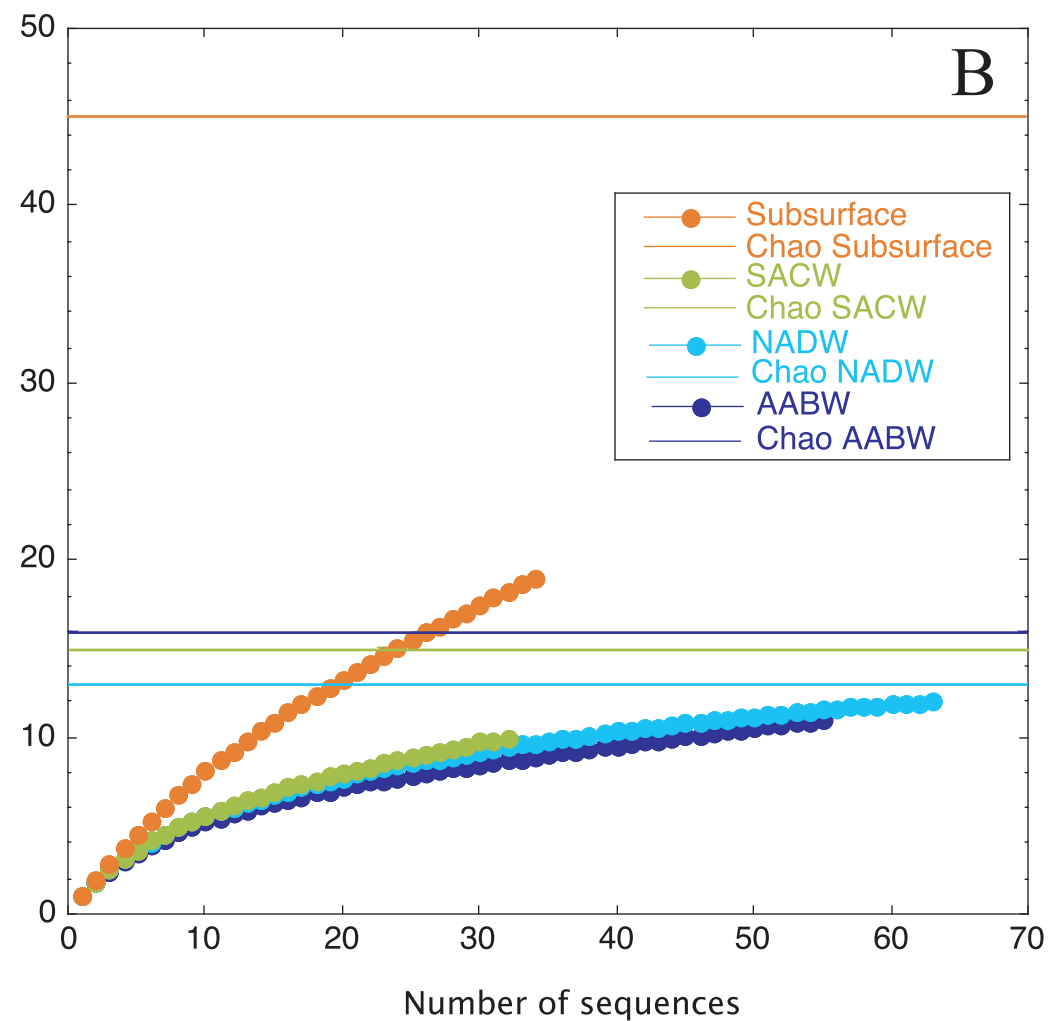

Sup.Fig.2

Supplement: Supplementary file 2 — Figure S2. Rarefaction analysis of the clone libraries of the bacterial (A) and archaeal (B) communities obtained from the different water masses at St. 12. The Chao index for the OTUs sharing 98% identity is indicated for each water mass by horizontal lines. [file fem0085-0537-sd2.pdf]

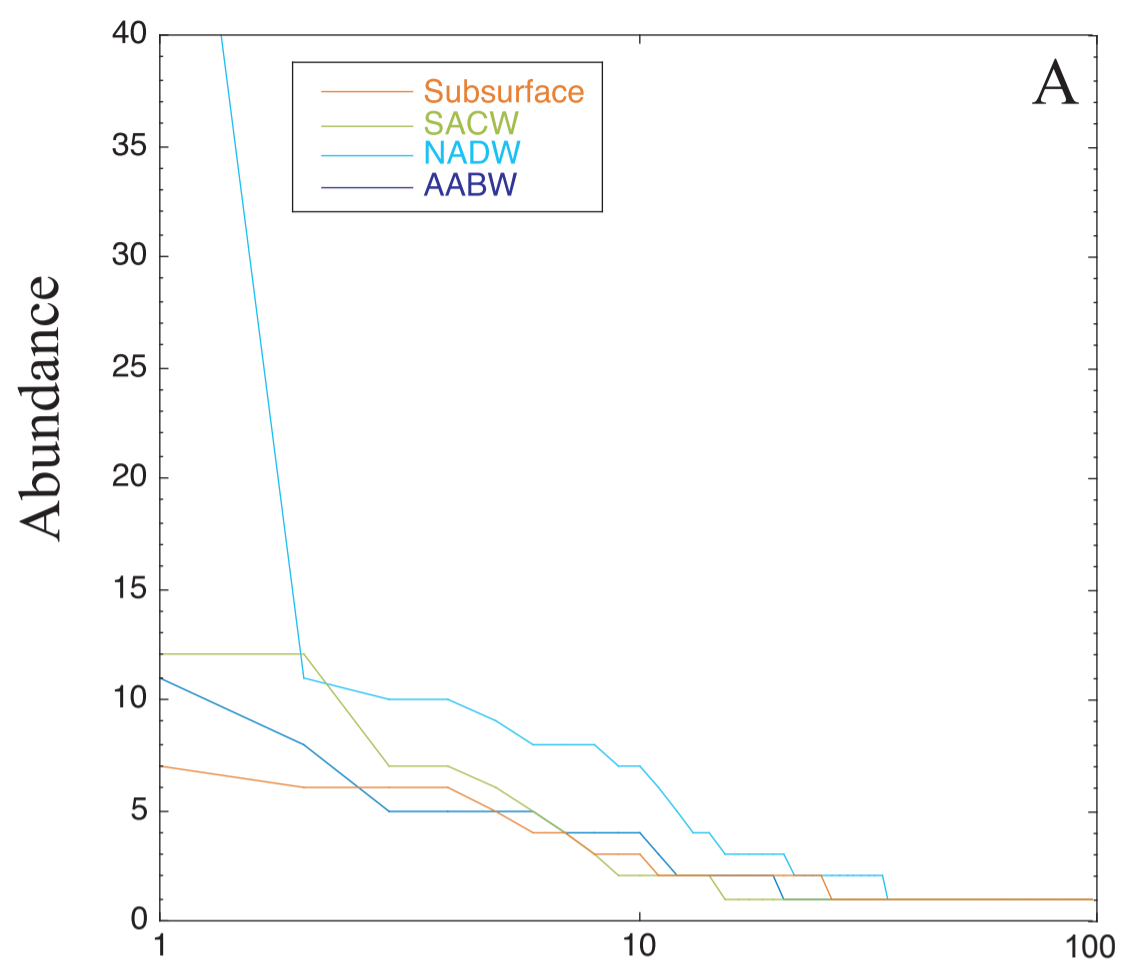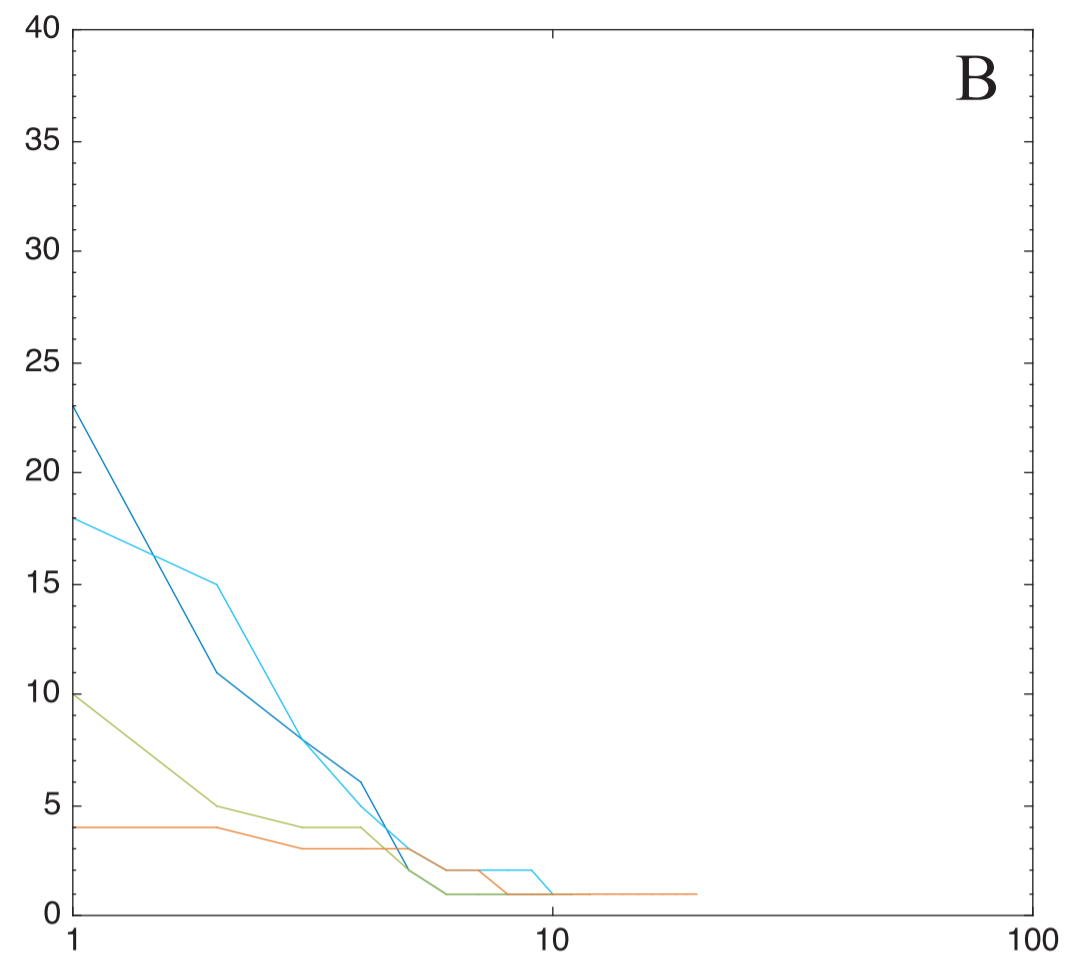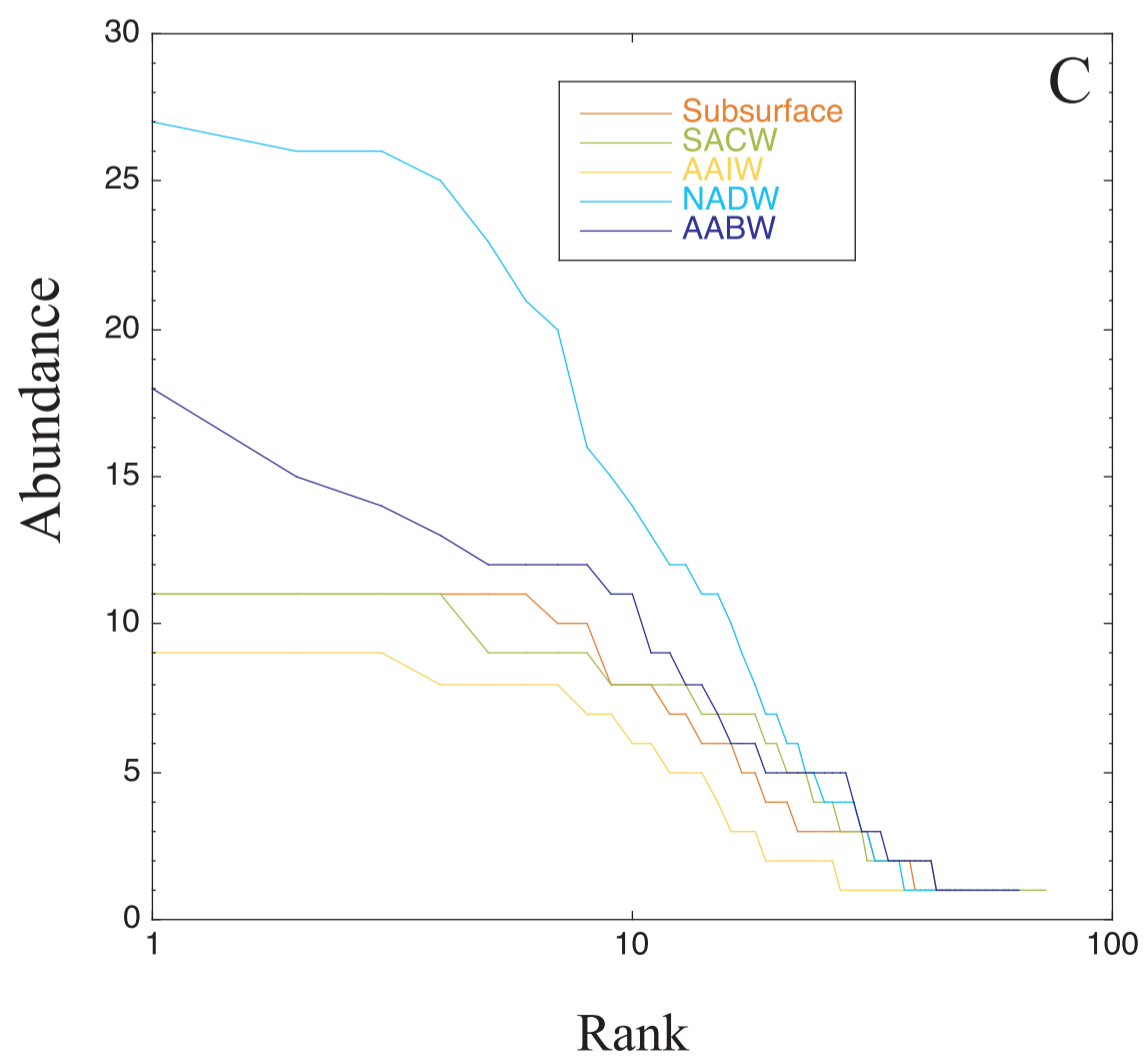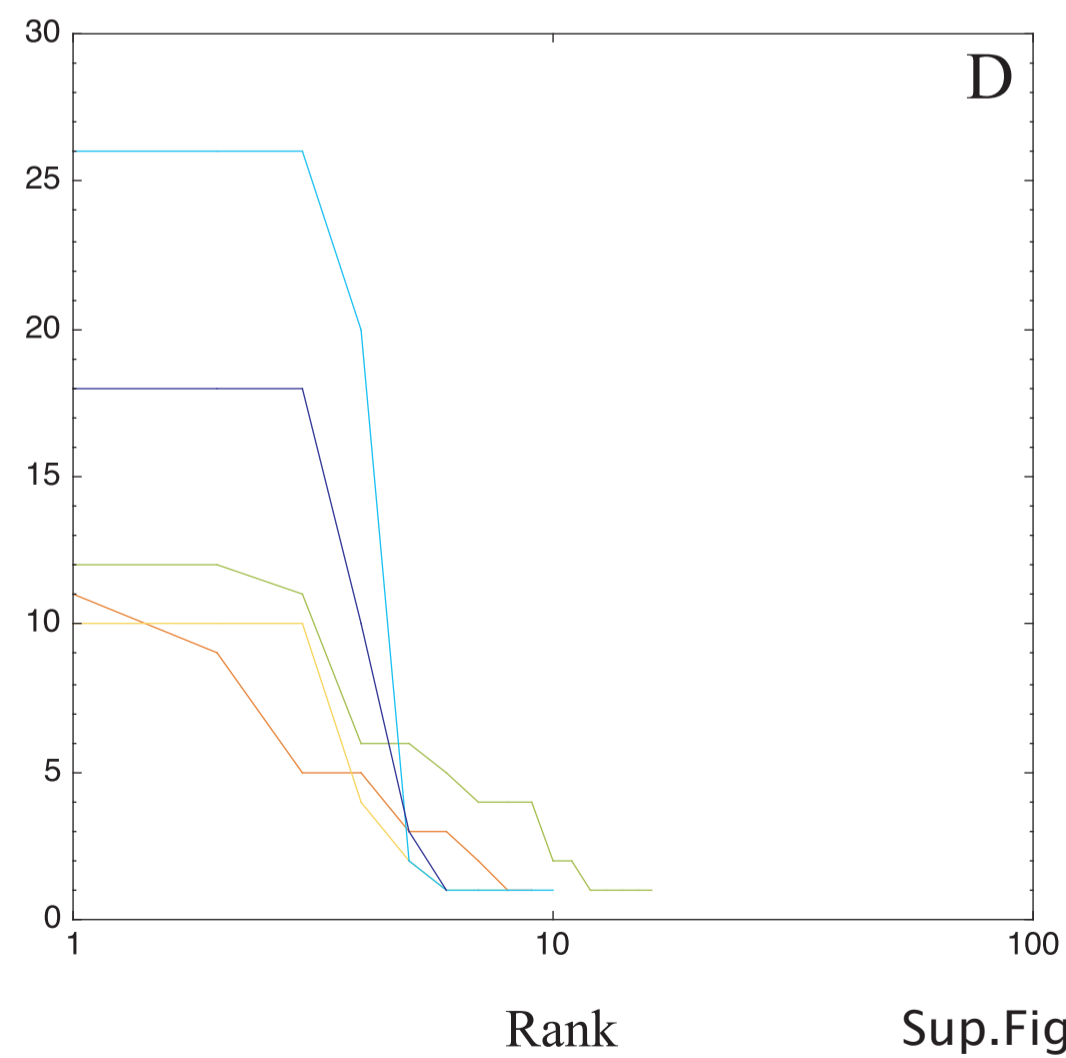

Supplement: Supplementary file 3 — Figure S3. Rank distribution of bacterial OTUs assessed by cloning and sequencing (A) and T-RFLP fingerprinting (C) and of archaeal OTUs assessed by cloning and sequencing (B) and T-RFLP fingerprinting (D). [file fem0085-0537-sd3.pdf]

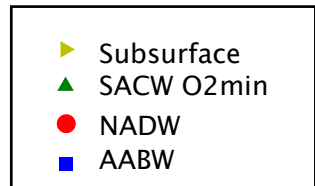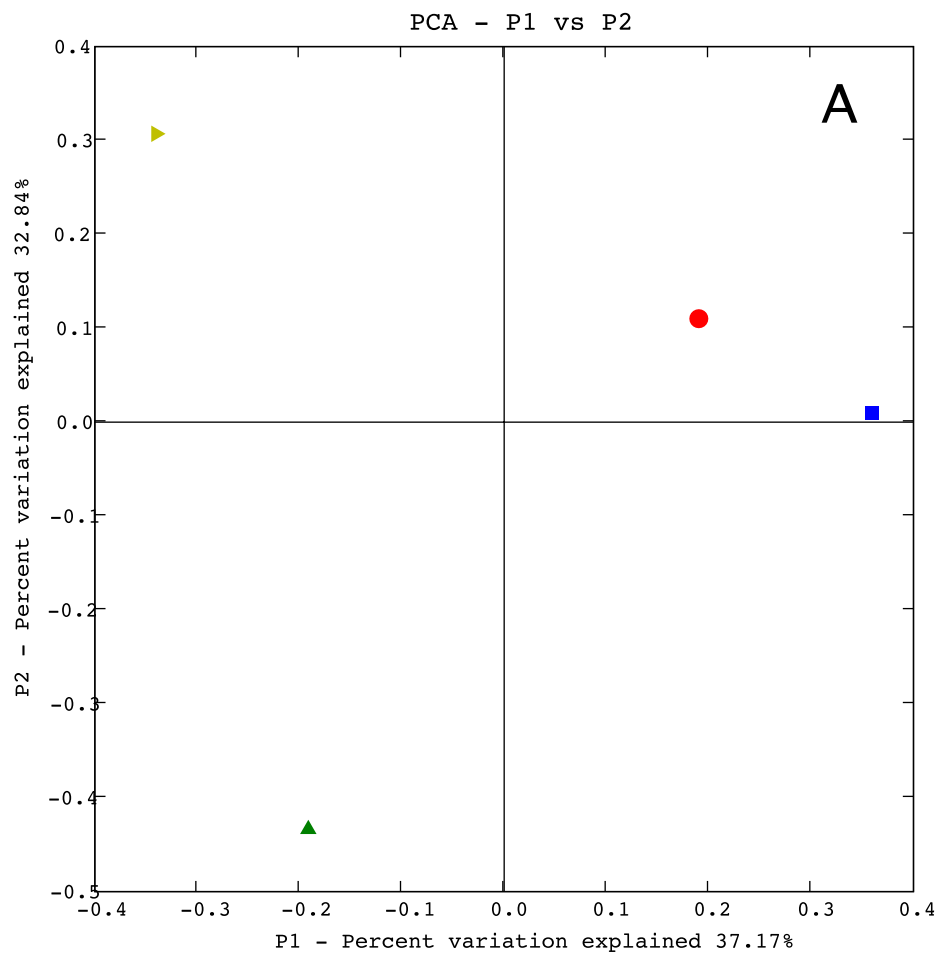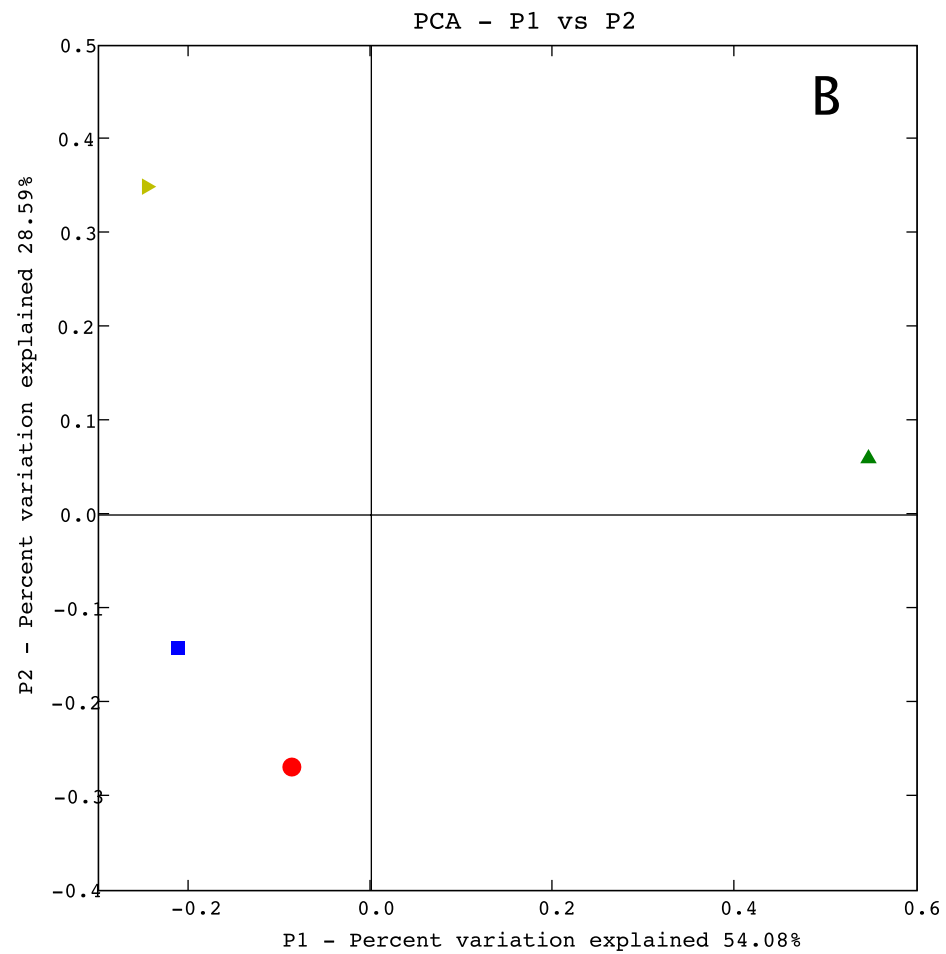

Supplement: Supplementary file 4 — Figure S4. Principal Component analysis of the bacterial (A) and archaeal (B) communities obtained along the Romanche Fracture Zone. [file fem0085-0537-sd4.pdf]

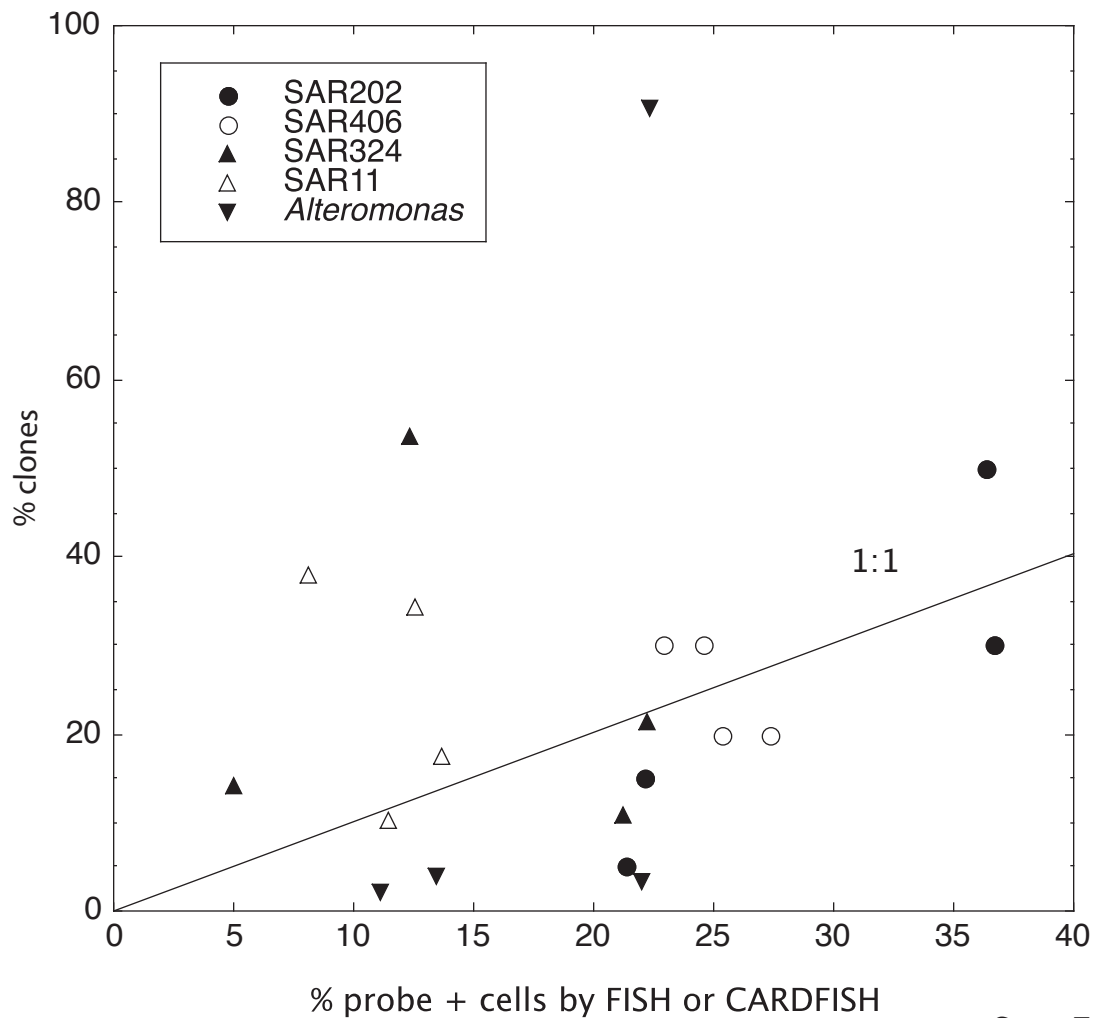

Supplement: Supplementary file 5 — Figure S5. Relative contribution of specific bacterial groups to the bulk bacterial abundance determined by FISH and CARD-FISH vs. the percentage of cells of the respective bacterial group retrieved by 16S rRNA gene clone libraries. [file fem0085-0537-sd5.pdf]
