# Supplementary material for: Spatial patterns of bacterial and archaeal communities along the Romanche Fracture Zone (tropical Atlantic)
Source: FEMS Microbiol Ecol. 2013 May 16;85(3):537–52. doi: 10.1111/1574-6941.12142 (PMC3840699; doi:10.1111/1574-6941.12142)
Supplement: Supplementary file 6 — Table S1. Overview of the probes and hybridization conditions applied in (CARD-)FISH analysis. [file fem0085-0537-sd6.docx]

**Table S1.** Overview of the probes and hybridization conditions applied in (CARD-)FISH analysis.

Probe Target organisms Sequence (5’-3’) % Formamide Reference

Eub338 Bacteria GCTGCCTCCCGTAGGAGT 55 Amann *et al*. (1990)

Eub338-II Supplement to Eub338 GCAGCCACCCGTAGGTGT 55 Daims *et al*. (1999)

Eub338-III Supplement to Eub338 GCTGCCACCCGTAGGTGT 55 Daims *et al*. (1999)

Cren554 Thaumarchaeota TTAGGCCCAATAATCMTCCT 20 Massana *et al*. (1997)

Cren 537 Thaumarchaeota TGACCACTTGAGGTGCTG 20 Teira *et al*. (2004)

SAR11-152 SAR11 ATTAG CACAAGTTTCCYCGTGT 45 Morris *et al*. (2002)

SAR11-441 SAR11 TACAGTCATTTTCTTCCCCGAC 45 Morris *et al*. (2002)

SAR11-542 SAR11 TCCGAACTACGCTAGGTC 45 Morris *et al*. (2002)

SAR11-732 SAR11 GTCAGTAATG ATCCAGAAAGYTG 45 Morris *et al*. (2002)

SAR202-104 SAR202 GTTACTCAGCCGTCTGCC 35 Morris *et al*. (2004)

SAR202-312 SAR202 TGTCTCAGTCCCCCTCTG 35 Morris *et a*l. (2004)

SAR324-1412 SAR324 GCCCCTGTCAACTCCCAT 35 Schattenhöfer *et al*. (2009)

SAR406-97 SAR406 CACCCGTTCGCCAGTTTA 65 Fuchs *et al*. (2005)

Alt1413 *Alteromonas, Colwellia* TTGCATCCCACTCCCAT 55 Eilers *et al*. (2000)

Amann RI, Binder BJ, Olson RJ, Chisholm SW, Devereux R & Stahl DA (1990) Combination of 16S rRNA-targeted oligonucleotide probes with flow cytometry for analyzing mixed microbial populations. *Appl Environ Microbiol* **56:** 1919–1925.

Daims H, Brühl A, Amann R, Schleifer KH & Wagner M (1999) The domain-specific probe EUB338 is insufficient for the detection of all Bacteria: Development and evaluation of a more comprehensive probe set. *Syst Appl Microbiol* **22:** 434–444.

Eilers H, Pernthaler J, Glöckner FO & Amann R (2000) Culturability and in situ abundance of pelagic bacteria from the North Sea. *Appl Environ Microbiol* **66:** 3044–3051.

Fuchs BM, Woebken D, Zubkov MV, Burkill PH & Amann R (2005) Molecular identification of picoplankton populations in contrasting waters of the Arabian Sea. *Aquat Microb Ecol* **39:** 145–157.

Massana R, Murray AE, Preston CM & De Long EF (1997) Vertical distribution and phylogenetic characterization of marine planktonic Archaea in the Santa Barbara Channel. *Appl Environ Microbiol* **63:** 50–56.

Morris RM, Rappe MS, Connon SA, Vergin KL, Siebold WA, Carlson CA & Giovannoni SJ (2002) SAR11 clade dominates ocean surface bacterioplankton communities. *Nature* **420:** 806–810

Schattenhöfer M, Fuchs BM, Amann R, Zubkov MV, Tarran G A & Pernthaler J (2009) Latitudinal distribution of prokaryotic picoplantkon populations in the Atlantic Ocean. *Environ Microbiol* **11:** 2078-2093.

Teira E, Reinthaler T, Pernthaler A, Pernthaler J & Herndl GJ (2004) Combining catalyzed reporter deposition-fluorescence in situ hybridization and microautoradiography to detect substrate utilization by Bacteria and Archaea in the deep ocean. *Appl Environ Microbiol* **70:** 4411–4414.
